# Evaluation of Antipsychotic-Induced Neuroleptic Malignant Syndrome Using a Self-Organizing Map

**DOI:** 10.7759/cureus.68260

**Published:** 2024-08-30

**Authors:** Koumi Miyasaka, Sakiko Hirofuji, Mika Maezawa, Satoshi Nakao, Moe Yamashita, Nanaka Ichihara, Yuka Nokura, Hirofumi Tamaki, Kazuhiro Iguchi, Mitsuhiro Nakamura

**Affiliations:** 1 Laboratory of Drug Informatics, Gifu Pharmaceutical University, Gifu, JPN; 2 Department of Pharmacy, Kyushu University Hospital, Fukuoka, JPN; 3 Laboratory of Community Pharmacy, Gifu Pharmaceutical University, Gifu, JPN

**Keywords:** self-organizing map, japanese adverse drug event report (jader) database, antipsychotic side effect, typical antipsychotic, atypical antipsychotic, neuroleptic malignant syndrome (nms)

## Abstract

Introduction

In neuropsychiatric pharmacotherapy, neuroleptic malignant syndrome (NMS) is a potentially serious side effect of antipsychotics characterized primarily by fever, disorientation, extrapyramidal disorders, and autonomic nervous system imbalance, which can lead to death if left untreated. We visualized the NMS profile of antipsychotics using a self-organizing map (SOM). We combined it with decision tree analysis to discriminate between 31 antipsychotics in more detail than typical antipsychotic (TAP) and atypical antipsychotic (AAP) classifications.

Method

A total of 20 TAPs and 11 AAPs were analyzed. We analyzed NMS reports extracted from the Japanese Adverse Drug Event Report (JADER) database based on standardized Medical Dictionary for Regulatory Activities (MedDRA) queries (Standardized MedDRA Queries (SMQ) code: 20000044, including 68 preferred terms). The SOM was applied using the SOM package in R version 4.1.2 (R Foundation for Statistical Computing, Vienna, Austria).

Results

The Japanese Adverse Drug Event Report (JADER) database contained 887,704 reports published between April 2004 and March 2024. The numbers of cases of NMS (SMQ code: 20000044) reported for risperidone, aripiprazole, haloperidol, olanzapine, and quetiapine were 1691, 1294, 1132, 1056, and 986, respectively. After the antipsychotics were classified into six units using SOM, they were adapted for decision tree analysis. First, 31 antipsychotics branched off into groups with loss of consciousness, with one group (10 TAPs) consisting entirely of TAPs, and the other consisting of antipsychotics that were further separated into two groups with coma induced by TAPs and AAPs.

Conclusion

The results of this study provide a reference for healthcare providers when predicting the NMS characteristics induced by each drug in patients, thereby facilitating the effective treatment of schizophrenia.

## Introduction

Neuroleptic malignant syndrome (NMS) is a rare and serious side effect that occurs with the use of antipsychotics and other drugs and is associated with symptoms such as pyrexia, altered state of consciousness, and extrapyramidal disorders such as muscle rigidity and tremor [1,2]. It is primarily caused by administration, reduction, or discontinuation of antipsychotic neuroleptics [1]. The frequency of NMS complications in patients taking antipsychotics is low (0.01%-3.2%) [3]. However, because NMS eventually becomes severe and potentially lethal, it should be detected early and treated appropriately [3]. Antipsychotics are also used in psychosomatic medicine, neurology, internal medicine, surgery, and anesthesiology [4]. Thus, NMS is not only relevant to the treatment of psychiatric problems but also affects health issues related to other disciplines [1-3].

The clinical manifestations of NMS include pyrexia and impaired consciousness, extrapyramidal disorders (muscle rigidity, tremor, dystonia, etc.), autonomic nervous system imbalance (hyperhidrosis, tachycardia, abnormal blood pressure, etc.), changes in mental status (altered state of consciousness, delirium, etc.), myoclonus, and respiratory failure [1]. In severe cases of NMS, lysis of skeletal muscle tissue occurs, leading to increased myoglobin levels in the blood and urine and acute renal failure [4]. In addition, NMS causes metabolic acidosis and disseminated intravascular coagulation [1]. Although the etiology and pathophysiology of NMS are not fully understood, many drugs, including antipsychotics and medications for Parkinson’s disease, may induce NMS and share dopamine receptor-blocking effects in the central nervous system [3,4]. Furthermore, the efficacy of dopaminergic agents such as bromocriptine in NMS supports the dopaminergic hypothesis of rapid and potent blockade of dopamine receptors in the substantia nigra and hypothalamus or dyscoordination of the dopaminergic nervous system with other monoamine nervous systems [1,3,4]. However, the diverse symptoms of NMS cannot be explained solely by the dopamine nervous system. The dopamine/serotonin nervous system imbalance hypothesis and effects of drug-metabolizing enzyme gene polymorphisms and neurotransmitter receptor gene polymorphisms have also been proposed. Moreover, risk factors on the part of the patient have been suggested but not proven with certainty, including physical factors such as dehydration, malnutrition, fatigue, infection, and concomitant organic brain disease; significant worsening of psychiatric symptoms such as stupor and psychomotor agitation; and history of NMS.

The development of NMS is suspected when multiple sudden changes in the autonomic nervous system, such as fever, sweating, and rapid changes in blood pressure, are observed after administration of a drug that acts on the psychoneurotic system. Currently, clinically used NMS diagnostic criteria include Levenson’s criteria [5] and Caroff’s criteria [1,6]. However, these criteria are for the definitive diagnosis of NMS and discriminate between hyperthyroidism (crisis), pheochromocytoma, dehydration, heat stroke, encephalitis, alcohol withdrawal symptoms, rhabdomyolysis, serotonin syndrome, fatal catatonia, etc.; they are not for distinguishing the circumstances in which antipsychotic drugs may cause NMS symptoms and are not intended to distinguish NMS symptoms caused by antipsychotic drugs.

There are no known early symptom characteristics of NMS, and the slow detection, severity, and unique course of onset remain matters of caution in medical practice. Owing to this difficulty in early intervention, there is a need for indicators to determine a variety of clinical symptoms. However, to our knowledge, information about these adverse events is scarce. Recently, the incidence profile of antipsychotic-induced NMS was comprehensively evaluated via cluster analysis, revealing 29 antipsychotic drugs and 52 adverse event profiles using the spontaneous reporting system (SRS) for adverse events published by the Japanese regulatory authorities [7]. Cluster analysis is a useful statistical technique in data mining that identifies natural groupings in a data set [8,9]. Hirofuji et al. classified antipsychotic drugs into three clusters and found information for each cluster [7]; however, the characterization involved only a simple classification of typical antipsychotics (TAPs) and atypical antipsychotics (AAPs), which is difficult to adapt to everyday clinical practice. Therefore, we believe that it is meaningful to propose new discriminative criteria that can account for the complex clinical manifestations of NMS.

Methods for classifying side-effect profiles include unsupervised learning techniques such as principal component analysis, cluster analysis, and self-organizing maps (SOMs). SOMs are a promising pattern recognition method first reported by Kohonen [10]. SOMs can cluster high-dimensional data without prior correct data [8-11], can automatically identify trends and correlations in large datasets, and efficiently compress large amounts of data. SOMs have been applied to visualize the side effects of anticancer agents and rhabdomyolysis [11,12] as they can be used to create easy-to-understand, visual, two-dimensional maps that reflect the data structure of adverse drug reaction information across all drugs with the same effect [13].

The SRS database is an accumulation of case reports of adverse events actually occurring in clinical practice and plays a major role in the safety evaluation of drugs [14]. The SRS uses competitive learning in SOMs to classify data into an optimal number of units and automatically acquires rules that explain the relationships between units using a tree structure. Visualization of adverse events using SOMs is expected to improve medical safety by tracking trends in the occurrence and avoidance of adverse drug events, prediction of new adverse drug reactions, and consideration of alternative drugs [13]. The purpose of this study was to classify NMS data for antipsychotic drugs into an optimal number of units using competitive learning with SOMs and to obtain rules that explain the relationship between NMSs using a tree structure.

## Materials and methods

Ethical approval

Ethical approval was not sought because the study was a database-related observational study without the direct involvement of any research subjects. All results were obtained from data openly available online from the PMDA website [15]. All data from the Japanese Adverse Drug Event Report (JADER) database were fully anonymized by the relevant regulatory authority before we accessed them.

Data source

The data source for adverse events was the Japanese Adverse Drug Event Report (JADER) database, which was collected and fully anonymized by the Pharmaceuticals and Medical Devices Agency (PMDA) and can be downloaded from the PMDA website [15]. The JADER data were divided into four files, namely, “demo,” “drug,” “reac,” and “hist,” and each data table was linked by an identification number and integrated into a relational database. The contents of each table were as follows: the “demo” table contained basic patient information such as gender, age, and reporting year; the “drug” table contained information about the drug administered, including generic name, route of administration, and start and end dates of administration; the “reac” table contained information about adverse events, outcomes, and onset dates; the “hist” table contained information about the patient’s primary disease.

The structure of this database is in accordance with the guidelines of the International Council on Harmonization E2B [16]. The “drugs” table listed the extent to which the drugs were presumed to be involved in adverse events as follows: “suspected drugs,” “concomitant drugs,” and “interacting drugs.” In this retrospective pharmacovigilance study, data on the “suspected drugs” were extracted and analyzed. We linked these four tables using identification numbers and integrated the relational databases using MySQL (Oracle, USA, version 8.0.36). Using this relational database system, we searched for the drug name in question and extracted case reports related to NMS for analysis.

Definition of adverse events

Adverse events in the JADER database were coded using the Medical Dictionary for Regulatory Activities (MedDRA) ver. 27.0 [17]. The Standardized MedDRA Queries (SMQ) database was used to analyze the SRSs. SMQs are groupings of MedDRA terms related to a defined condition or area of interest, usually at the preferred term (PT) level [17]. We used SMQs related to NMS (SMQ code: 20000044, including 68 PTs) (Table 1).

**Table 1:** Number of adverse events for each preferred term associated with neuroleptic malignant syndrome by antipsychotics. Source: Reference [17].

Preferred terms	Preferred term code	Case (n)	Total (n)	Reporting ratio (RR) (%)
Altered state of consciousness	10001854	460	7,630	6.03
Autonomic nervous system imbalance	10003840	3	126	2.38
Blood creatine phosphokinase abnormal	10005468	2	13	15.38
Blood creatine phosphokinase increased	10005470	496	3,748	13.23
Blood creatine phosphokinase MM increased	10005477	0	1	0.00
Blood pressure abnormal	10005728	1	54	1.85
Blood pressure decreased	10005734	154	10,327	1.49
Blood pressure fluctuation	10005746	7	137	5.11
Blood pressure increased	10005750	51	4,125	1.24
Body temperature increased	10005911	3	129	2.33
Catatonia	10007776	48	77	62.34
Coma	10010071	85	540	15.74
Confusional state	10010305	36	491	7.33
Delirium	10012218	225	3,639	6.18
Depressed level of consciousness	10012373	261	4,154	6.28
Disorientation	10013395	9	409	2.20
Dyskinesia	10013916	230	1,181	19.48
Dystonia	10013983	582	769	75.68
Extrapyramidal disorder	10015832	313	553	56.60
Heart rate abnormal	10019300	0	11	0.00
Heart rate increased	10019303	23	689	3.34
Hyperhidrosis	10020642	34	852	3.99
Hyperkinesia	10020651	11	38	28.95
Hyperpyrexia	10020741	3	74	4.05
Hypertension	10020772	52	4,064	1.28
Hyperthermia malignant	10020844	5	329	1.52
Hypertonia	10020852	4	37	10.81
Hypotension	10021097	88	2,867	3.07
Labile blood pressure	10023533	2	25	8.00
Leukocytosis	10024378	3	88	3.41
Loss of consciousness	10024855	232	7,187	3.23
Muscle necrosis	10028320	0	27	0.00
Muscle rigidity	10028330	58	196	29.59
Myoclonus	10028622	28	388	7.22
Myoglobin blood increased	10028625	3	58	5.17
Myoglobinuria	10028629	2	34	5.88
Myoglobin urine present	10028631	5	11	45.45
Neuroleptic malignant syndrome	10029282	2,758	3,961	69.63
Oculogyric crisis	10030071	167	226	73.89
Opisthotonus	10030899	3	14	21.43
Parkinsonism	10034010	417	930	44.84
Pyrexia	10037660	268	20,897	1.28
Rhabdomyolysis	10039020	932	7,703	12.10
Serotonin syndrome	10040108	96	879	10.92
Slow response to stimuli	10041045	1	12	8.33
Stupor	10042264	62	157	39.49
Tachycardia	10043071	99	2,104	4.71
Tremor	10044565	126	1,798	7.01
Unresponsive to stimuli	10045555	8	131	6.11
White blood cell count abnormal	10047940	0	23	0.00
White blood cell count increased	10047943	38	1,239	3.07
Parkinsonian crisis	10048868	0	5	0.00
Labile hypertension	10049079	0	1	0.00
Consciousness fluctuating	10050093	2	16	12.50
Parkinsonian rest tremor	10056437	0	0	–
Muscle enzyme increased	10057945	1	16	6.25
Myoglobinaemia	10058735	0	21	0.00
Myoglobin blood present	10059888	0	1	0.00
Freezing phenomenon	10060904	2	6	33.33
Parkinson's disease	10061536	57	489	11.66
Cardiovascular insufficiency	10065929	3	32	9.38
Resting tremor	10071390	0	13	0.00
Hyporesponsive to stimuli	10071552	0	33	0.00
Dystonic tremor	10073210	0	0	–
Necrotizing myositis	10074769	0	18	0.00
Malignant catatonia	10080149	27	34	79.41
Withdrawal catatonia	10081010	3	5	60.00
Muscle enzyme abnormal	10087463	0	0	–

In this study, we first searched for PTs of 68 adverse events; 14 PTs were not reported, and so 54 adverse events were used in subsequent analyses.

Target drugs

We analyzed 20 TAPs and 11 AAPs based on the “Schizophrenia Medication Guide for Patients and Supporters 2022” (Table 2) [18].

**Table 2:** Number of adverse events associated with neuroleptic malignant syndromes of antipsychotics and unit number assigned to the 6 × 1 map created by self-organization map. ^a^Source: Reference [18]. ^b^TAP: typical antipsychotic. ^c^AAP: atypical antipsychotic.

Drug name^a^	Case (n)	Total (n)	Reporting ratio (%)	Unit
Bromperidol^b^	85	173	49.13	4
Chlorpromazine^b^	651	1,656	39.31	3
Clocapramine^b^	11	25	44.00	5
Fluphenazine^b^	74	133	55.64	4
Haloperidol^b^	1,132	2,099	53.93	1
Levomepromazine^b^	548	1,266	43.29	3
Mosapramine^b^	12	26	46.15	5
Nemonapride^b^	5	16	31.25	5
Oxypertine^b^	6	18	33.33	5
Perphenazine^b^	51	99	51.52	1
Pimozide^b^	25	61	40.98	5
Pipamperone^b^	7	21	33.33	4
Prochlorperazine^b^	87	236	36.86	2
Propericiazine^b^	60	132	45.45	3
Reserpine^b^	4	18	22.22	5
Spiperone^b^	1	2	50.00	5
Sultopride^b^	53	86	61.63	5
Tiapride^b^	199	485	41.03	2
Timiperone^b^	18	41	43.90	5
Zotepine^b^	231	485	47.63	4
Aripiprazole^c^	1,294	4,026	32.14	2
Asenapine^c^	158	551	28.68	3
Blonanserin^c^	496	1,148	43.21	3
Brexpiprazole^c^	212	587	36.12	3
Clozapine^c^	482	3,613	13.34	3
Lurasidone^c^	155	598	25.92	4
Olanzapine^c^	1,056	3,228	32.71	3
Paliperidone^c^	443	1,733	25.56	4
Perospirone^c^	280	593	47.22	3
Quetiapine^c^	986	3,444	28.63	3
Risperidone^c^	1,691	4,461	37.91	2

SOM

A SOM is a feedforward neural network model that uses an unsupervised learning algorithm. It compresses information from multidimensional data and draws low-dimensional (often two-dimensional) maps. It flows from the first layer (input layer) to the second layer (output layer) in one direction and never returns. SOM is based on competitive learning, where each neuron competes for similarity to the input data. The neuron with the highest similarity to the input data is the winner and wins all rights to learn that data. In addition, the weights of neurons in the winner’s neighborhood are also updated, placing neurons with higher similarity closer together and neurons with lower similarity farther apart [10]. When using data mining methods such as SOM, it is generally important to consider all adverse events without omissions. In this study, a two-dimensional table depicting the percentages of all reported adverse events related to NMS and a list of drugs was created (see Table 3-Table 6 in the Appendices). Subsequently, 54 adverse events, which were placed in layer 1 (input layer), and 100 units, which was sufficiently greater than the number of drugs (31 drugs), were placed in a 10 × 10 format in layer 2 (output layer) for competitive learning.

The SOM was performed using the SOM package in R version 4.1.2 (R Foundation for Statistical Computing, Vienna, Austria). Rpart is a machine-learning library in R used to build classification and regression trees. Decision tree analysis was performed using the rpart package.

## Results

**Figure 1:**
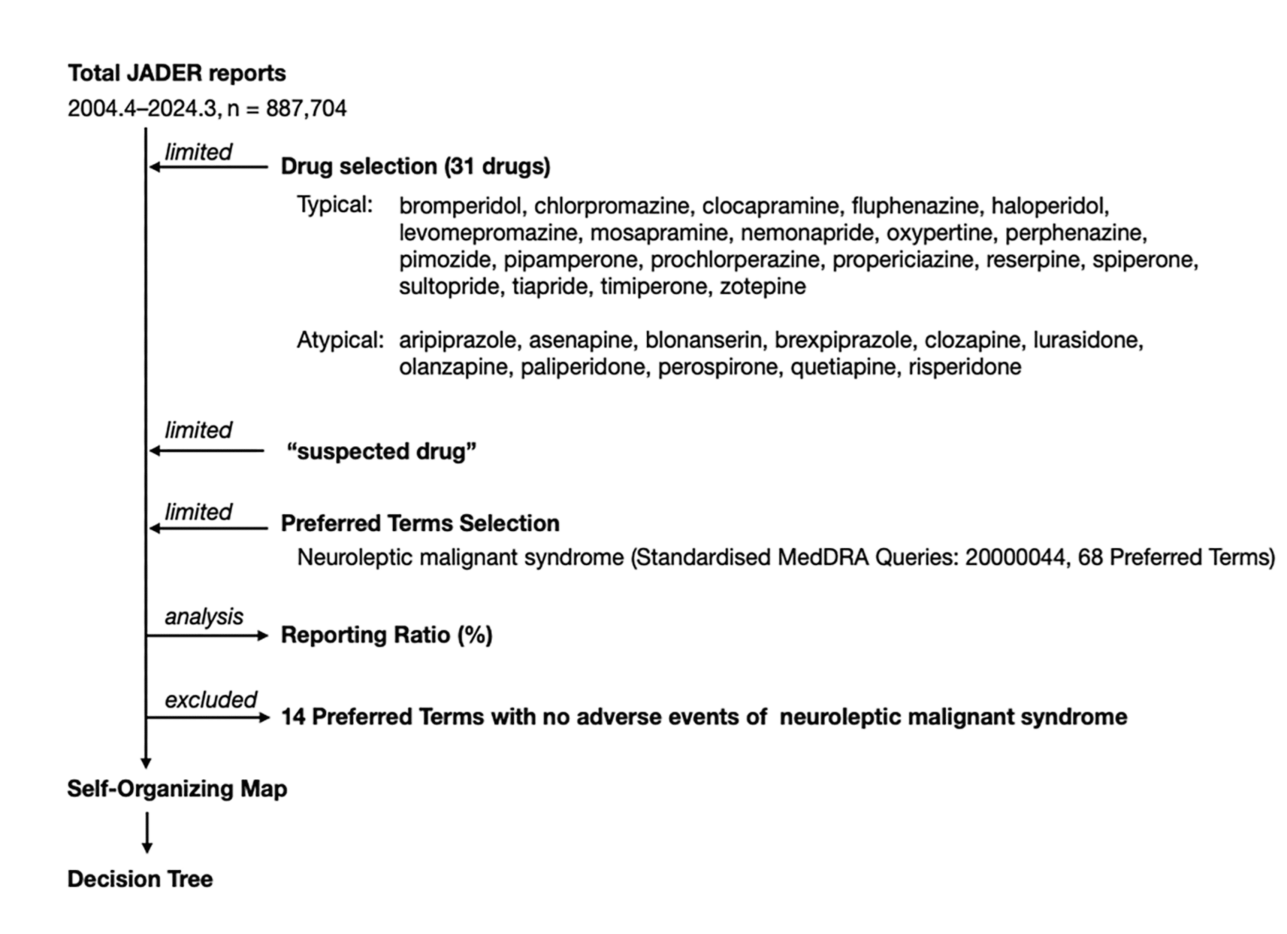
Flowchart of data analysis. JADER: Japanese Adverse Drug Event Report.

The JADER database contained 887,704 reports published between April 2004 and March 2024 (Figure 1).

**Figure 2:**
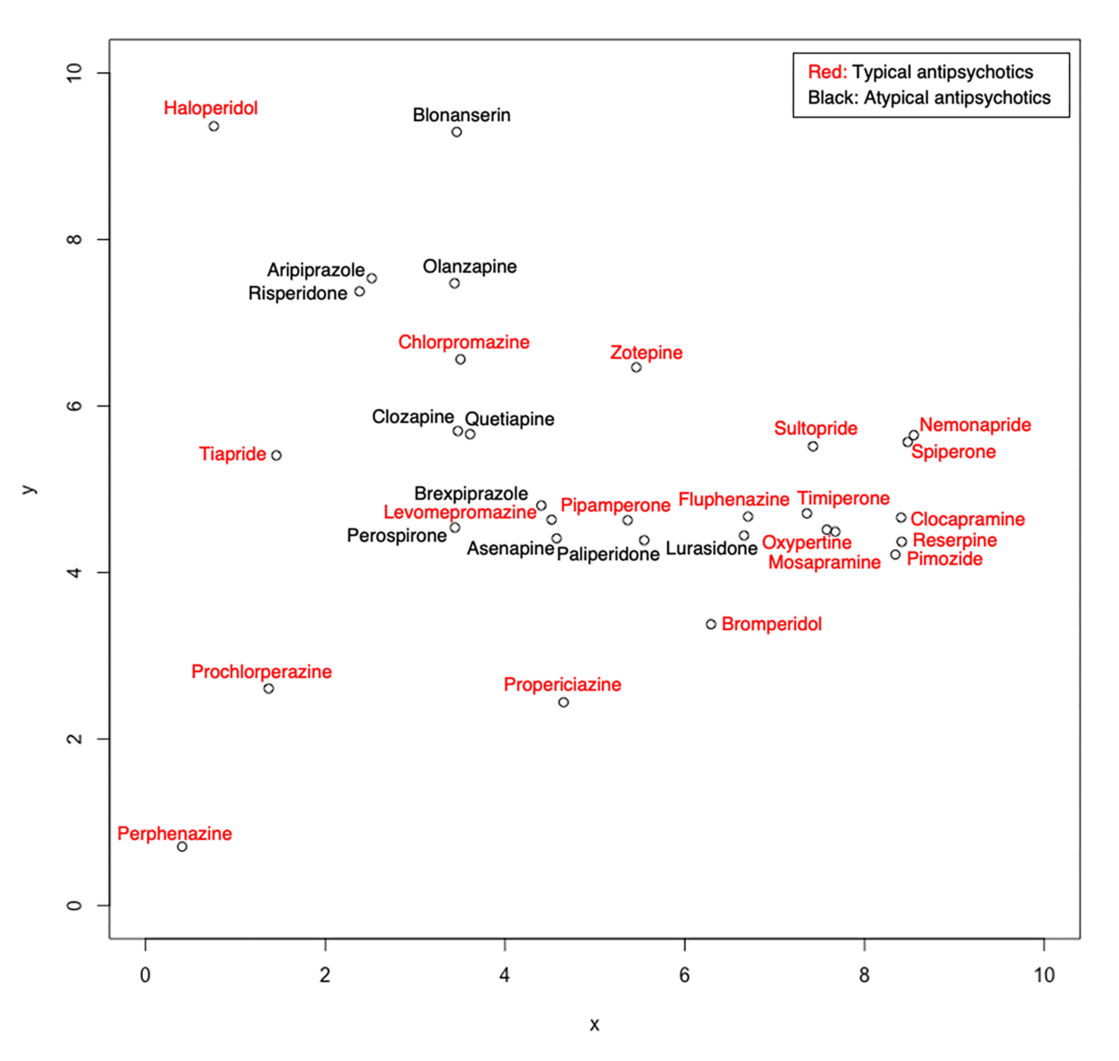
Self-organization map of 31 antipsychotics by neuroleptic malignant syndromes.

The number of cases of NMS (SMQ code: 20000044) reported for risperidone, aripiprazole, haloperidol, olanzapine, and quetiapine was 1691, 1294, 1132, 1056, and 986, respectively (Table 2). The NMS reporting ratios (RRs) for each antipsychotic are summarized in Table 3-Table 6 of the Appendices. The SOM results are shown as a 10 × 10 positioning map in Figure 2.

**Figure 3:**
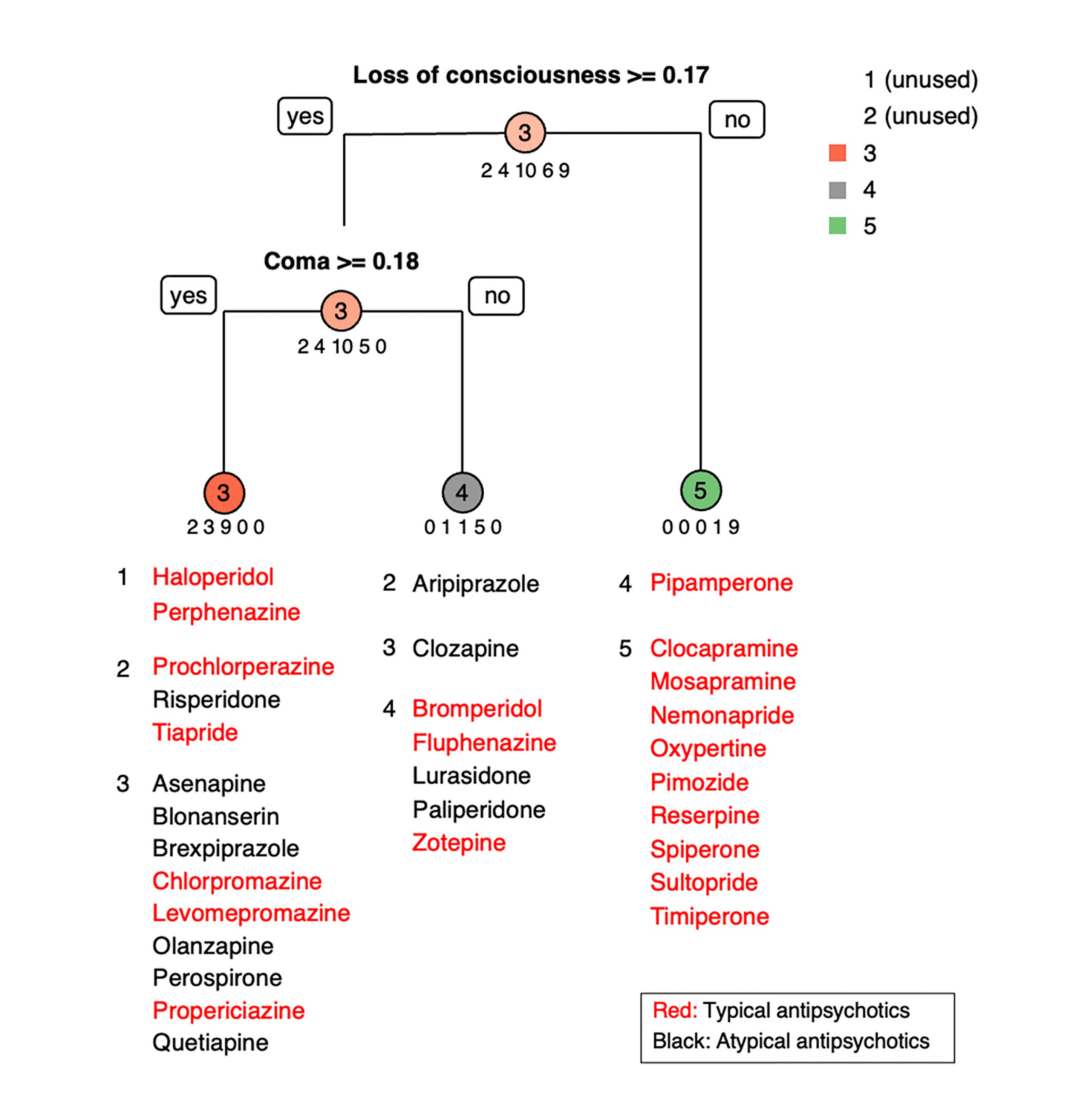
Decision tree of 31 antipsychotics by neuroleptic malignant syndromes.

The adjacent units in the SOM observation map were more similar to the distant units. However, the interpretation of clinically meaningful units is difficult. Therefore, the number of units in the first layer remained at 54, and the number of units in the second layer was set to 6, which were arranged in a 6 × 1 map for competitive learning. The decision tree grew with the unit number assigned to the SOM as the criterion variable and the 54 adverse events as predictors (Table 2, Figure 3).

The 31 antipsychotics branched into unit 5 (clocapramine, mosapramine, nemonapride, oxypertine, etc.) if the RR for loss of consciousness was less than 0.17%. Antipsychotics with a loss of consciousness RR of 0.17% or greater were further divided based on the RR for coma, with drugs having RR for coma at less than 0.18% in unit 4 (bromperidol, fluphenazine, lurasideone, etc.) and those with more than 0.18% in unit 3 (asenapine, chlorpromazine, levomepromazine, olanzapine, quetiapine, etc.).

## Discussion

In this study, we visualized the NMS profile of antipsychotics using a SOM and combined it with decision tree analysis to discriminate between 31 antipsychotics in more detail than the current TAP and AAP classifications permit.

A SOM is a mathematical model of brain circuits that extracts the essence of how functional maps of the sensory cortex are formed by learning based on perceptual experience; the algorithm for information processing (competitive learning) was proposed by Kohonen in 1982 [10]. This model enables the creation of two-dimensional maps that can visualize high-dimensional datasets. From the perspective of statistical analysis, it can be regarded as an extension of principal component analysis, a linear method, to nonlinear cases. In addition to SOM, other methods for compressing multidimensional information and drawing low-dimensional maps in the traditional field of multivariate analysis include principal component, factor, and discriminant analyses. However, these methods have limitations. For example, two-dimensional principal component analysis can only consider two major features and information that cannot be represented is discarded. In contrast, in a SOM, if the number of units is increased and the map is widened, the differences between observations can be represented in as much detail as possible. In general, antipsychotics have been reported to pose a risk of NMS associated with standard doses and routes of administration. All the antipsychotics included in the current study have been reported to cause some form of NMS. In the SOM shown in Figure 1, the adjacent units are more similar than the distant units. Therefore, drugs with different NMS profiles are placed far apart from each other.

Antipsychotics are classified as either TAPs or AAPs. TAPs have a very high affinity for dopamine D2 receptors and are generally effective for positive symptoms such as delusions and hallucinations, whereas AAPs exhibit serotonin 5-HT2 receptor antagonist activity in addition to dopamine D2 receptor antagonist activity and are effective for negative symptoms such as emotional numbing and lack of spontaneity [19,20]. Although the clinical manifestations of NMS are diverse, its etiology and pathogenesis have not yet been fully elucidated, and most NMS symptoms are thought to result from a rapid decrease in central dopaminergic activity due to the blockade of D2 receptors or abrupt cessation of D2 receptor stimulation [4]. In this regard, the higher the dose of dopamine receptor inhibitors administered, the greater the risk of developing NMS. However, dosage was not incorporated into the present analysis and is a subject for future work.

AAPs are currently the treatment of choice for first-episode schizophrenia [21]. Previous reports suggest that the risk of NMS with AAPs is relatively low compared with that with TAPs [22]. A balanced blockade of dopamine D2 and 5-HT receptors, especially 5-HT2A receptors, in AAPs has been proposed, albeit inconclusively, as a reason for the lower risk of NMS with AAPs than with TAPs [23].

Units 1 and 5 in Figure 3 consisted only of TAPs, and all TAPs in unit 5 are clustered on the right side of Figure 2. Drugs with similar patterns of adverse events are placed closer together in the SOM, suggesting that the adverse event patterns of TAPs within unit 5 are similar. These results provide a reference for healthcare providers when identifying adverse events and determining appropriate responses. On the other hand, it is interesting to note from Figure 2 and Figure 3 that TAP and AAP are mixed in units 2-4. No established theory compares TAP- and AAP-related NMS risks as indicated by the distribution of TAPs from units 2 to 4 (Figure 3). However, the results show that clinicians should not view adverse events of psychotic drugs in units 2-4 as simply categorized as TAP and AAP. On a SOM for antimicrobial agents, Kawakami et al. found that drugs for which new adverse events were reported were adjacent to drugs for which adverse events had already been observed [13], indicating that SOMs can predict the occurrence of new adverse events to some extent and may be useful for early risk avoidance [13]. Thus, similar new adverse events may be reported for drugs in close proximity in Figure 2.

Owing to the cluster analysis of the previous study classified the data into three clusters [7], we considered the number of units that branch twice in the decision tree to be a classification that is easily applicable to clinicians. In this study, the units obtained in the 6 x 1 map gave two branches in the decision tree. The structure of the decision tree remained almost unchanged when the number of in the second layer was increased to six or more (data not shown). The number of SOM units used in the decision tree is based on subjectivity or a point of view and can be divided into arbitrary numbers by the researcher. The validity of SOM results can only be subsequently determined through external knowledge. If the user can interpret the decision tree result, then it can be said that a meaningful number of units has been formed. Therefore, the validity of SOM results must always be considered based on the purpose of their use.

Patients receiving AAPs are less likely to experience extrapyramidal disturbances than those receiving TAPs [24]. However, several previous studies have shown that AAPs can cause extrapyramidal disturbances to the same extent as TAPs [25,26]. Association rule mining by Hirofuji et al. showed that AAP-related rules were more common than TAP-related rules for extrapyramidal disorders [7]. Interestingly, the first branch of the decision tree in Figure 3 represents the percentage of reported cases of loss of consciousness, whereas the next branch represents the percentage of reported cases of coma. NMS is a known precipitant of diabetic coma, including hyperosmotic hyperglycemic states. This potential interaction can be fatal if not detected early [27-29]. This finding suggests that the clinical manifestations of antipsychotic-induced NMS may be divided into the appearance of loss of consciousness and coma, which may help healthcare providers detect NMS earlier.

The limitations of analyses using the SRS include under- and over-reporting, the presence of confounding factors, insufficient information on patient background, the possibility of overlooking late adverse events, and a lack of a control or reference population. Given these inherent problems, SRSs should not be used for true risk assessment [14]. The possibility of publication bias was also noted for NMS. Although polypharmacy has been shown to be a risk factor for NMS [30], it was not adequately considered in this analysis. Indications and doses of each antipsychotic were also not considered. Frequent intramuscular injections are considered a risk factor for NMS [1], but the method of administration was not considered in this analysis. Future studies should consider these factors.

Patient information (disease name, age, gender, etc.) in the JADER dataset can be used not only to classify NMS, but also to estimate patients who are likely to develop a particular NMS. Utilization of this information is a future challenge. Although only JADER data were used in this analysis, it would be possible to create a more reliable SOM by integrating and analyzing other data, such as “Medical Information Datasets and the National Database of Health Insurance Claims and Specific Health Checkups of Japan” and FDA Adverse Event Reporting System (FAERS), the SRS of the U.S.

Despite the analytical limitations of the SRS used in this study, the findings using JADER, which has accumulated real-life clinical cases, are useful given the difficulty in conducting clinical trials to evaluate the diverse adverse event profiles of NMS. Continued medication is important for the treatment of schizophrenia. However, because treatment with antipsychotic medications extends over a long term, many patients discontinue medications at their discretion. The results of this study will help healthcare providers to predict the NMS characteristics that each drug is likely to induce in patients, thereby contributing to effective schizophrenia treatment. Furthermore, the usefulness of adverse event SOMs, which can visualize the similarity of diverse and complex adverse events of pharmaceuticals in a comprehensive two-dimensional manner, and the usefulness of the discriminant method combined with decision tree analysis were confirmed.

## Conclusions

The complex NMS profile elucidated using the SRS data was classified using a SOM, and each unit was characterized using decision tree analysis. The clinical manifestations of antipsychotic-induced NMS may be divided into the appearance of loss of consciousness and coma, which may help healthcare providers to detect NMS earlier. The results of this study may be useful in the management of NMS using antipsychotic medications.

## Appendices

**Table 3:** Reporting ratios (%) of preferred term of neuroleptic malignant syndrome associated with antipsychotics. ^a^Source: References [17, 18] ^b^TAP: typical antipsychotic ^c^AAP: atypical antipsychotic

Preferred term^a^	Preferrd term code^b^	Bromperidol^b^	Chlorpromazine^b^	Clocapramine^b^	Fluphenazine^b^	Haloperidol^b^	Levomepromazine^b^	Mosapramine^b^
Altered state of consciousness	10001854	0.0000	3.2609	4.0000	2.2556	1.4293	3.3175	3.8462
Autonomic nervous system imbalance	10003840	0.0000	0.0000	0.0000	0.0000	0.0000	0.0000	0.0000
Blood creatine phosphokinase abnormal	10005468	0.0000	0.0000	0.0000	0.0000	0.0000	0.0000	0.0000
Blood creatine phosphokinase increased	10005470	0.5780	1.8720	20.0000	1.5038	2.0962	1.8167	0.0000
Blood pressure abnormal	10005728	0.0000	0.0000	0.0000	0.0000	0.0476	0.0000	0.0000
Blood pressure decreased	10005734	0.0000	0.4227	4.0000	0.0000	0.8576	0.8689	0.0000
Blood pressure fluctuation	10005746	0.0000	0.0000	0.0000	0.0000	0.0953	0.0000	0.0000
Blood pressure increased	10005750	0.0000	0.1208	0.0000	0.0000	0.1429	0.0000	0.0000
Body temperature increased	10005911	0.0000	0.0000	0.0000	0.0000	0.0953	0.0000	0.0000
Catatonia	10007776	0.0000	0.3623	0.0000	0.0000	0.8576	0.2370	0.0000
Coma	10010071	0.0000	0.6643	0.0000	0.0000	0.1906	0.7109	0.0000
Confusional state	10010305	0.5780	0.2415	0.0000	0.0000	0.0953	0.3949	0.0000
Delirium	10012218	0.0000	0.6643	0.0000	0.0000	0.6670	0.8689	0.0000
Depressed level of consciousness	10012373	0.0000	0.5435	0.0000	0.7519	1.7151	0.7109	0.0000
Disorientation	10013395	0.0000	0.0000	0.0000	0.0000	0.1429	0.0000	0.0000
Dyskinesia	10013916	4.0462	1.5097	4.0000	6.0150	1.7627	1.3428	3.8462
Dystonia	10013983	12.1387	3.3213	8.0000	2.2556	4.0495	4.2654	0.0000
Extrapyramidal disorder	10015832	2.3121	0.7246	0.0000	2.2556	2.1915	0.7109	0.0000
Heart rate increased	10019303	0.0000	0.0604	0.0000	0.0000	0.0953	0.0790	0.0000
Hyperhidrosis	10020642	0.0000	0.0000	0.0000	0.0000	0.3335	0.1580	0.0000
Hyperkinesia	10020651	0.0000	0.0000	0.0000	0.0000	0.0000	0.0000	0.0000
Hyperpyrexia	10020741	0.0000	0.0000	0.0000	0.0000	0.0476	0.0000	0.0000
Hypertension	10020772	0.0000	0.0000	0.0000	0.0000	0.0476	0.0790	0.0000
Hyperthermia malignant	10020844	0.0000	0.0604	0.0000	0.0000	0.0476	0.0790	0.0000
Hypertonia	10020852	0.0000	0.0000	0.0000	0.0000	0.0953	0.0000	0.0000
Hypotension	10021097	1.7341	0.3019	8.0000	0.0000	0.0953	1.0269	0.0000
Labile blood pressure	10023533	0.0000	0.0000	0.0000	0.0000	0.0000	0.0000	0.0000
Leukocytosis	10024378	0.0000	0.0000	0.0000	0.0000	0.0000	0.0000	0.0000
Loss of consciousness	10024855	0.5780	0.8454	0.0000	0.7519	0.3811	1.0269	0.0000
Muscle rigidity	10028330	0.0000	0.0000	0.0000	0.0000	0.3335	0.2370	0.0000
Myoclonus	10028622	0.0000	0.1208	0.0000	0.0000	0.0476	0.0000	0.0000
Myoglobin blood increased	10028625	0.0000	0.0604	0.0000	0.0000	0.0476	0.0000	0.0000
Myoglobinuria	10028629	0.0000	0.0000	0.0000	0.0000	0.0000	0.0000	0.0000
Myoglobin urine present	10028631	0.0000	0.0000	0.0000	0.0000	0.0476	0.0000	0.0000
Neuroleptic malignant syndrome	10029282	23.6994	17.7536	4.0000	34.5865	31.3959	20.0632	34.6154
Oculogyric crisis	10030071	0.5780	0.0604	0.0000	0.0000	0.5241	0.2370	0.0000
Opisthotonus	10030899	0.0000	0.0000	0.0000	0.0000	0.0000	0.0000	0.0000
Parkinsonism	10034010	2.3121	1.8720	0.0000	4.5113	2.6679	2.1327	3.8462
Pyrexia	10037660	0.5780	1.3285	0.0000	0.7519	1.1434	1.1058	0.0000
Rhabdomyolysis	10039020	2.3121	6.5821	0.0000	7.5188	6.7651	7.0300	0.0000
Serotonin syndrome	10040108	0.0000	0.2415	0.0000	0.0000	0.0476	0.2370	0.0000
Slow response to stimuli	10041045	0.0000	0.0000	0.0000	0.0000	0.0000	0.0000	0.0000
Stupor	10042264	0.5780	0.3019	0.0000	0.0000	0.1906	0.3160	0.0000
Tachycardia	10043071	0.0000	0.1208	0.0000	0.0000	0.4764	0.3160	0.0000
Tremor	10044565	0.0000	0.4227	0.0000	1.5038	0.7146	0.5529	0.0000
Unresponsive to stimuli	10045555	0.0000	0.0604	0.0000	0.0000	0.0476	0.0000	0.0000
White blood cell count increased	10047943	0.0000	0.1208	0.0000	0.0000	0.1429	0.1580	0.0000
Consciousness fluctuating	10050093	0.0000	0.0000	0.0000	0.0000	0.0000	0.0000	0.0000
Muscle enzyme increased	10057945	0.0000	0.0000	0.0000	0.0000	0.0000	0.0000	0.0000
Freezing phenomenon	10060904	0.0000	0.0000	0.0000	0.0000	0.0000	0.0000	0.0000
Parkinson's disease	10061536	0.5780	0.0000	0.0000	0.0000	0.2382	0.2370	0.0000
Cardiovascular insufficiency	10065929	0.0000	0.0000	0.0000	0.0000	0.0000	0.0000	0.0000
Malignant catatonia	10080149	0.0000	0.7850	0.0000	0.0000	0.0476	0.0000	0.0000
Withdrawal catatonia	10081010	0.0000	0.0000	0.0000	0.0000	0.0000	0.0000	0.0000

**Table 4:** Reporting ratios (%) of preferred term of neuroleptic malignant syndrome associated with antipsychotic (continued). ^a^Source: References [17, 18] ^b^TAP: typical antipsychotic ^c^AAP: atypical antipsychotic

Preferred term^a^	Nemonapride^b^	Oxypertine^b^	Perphenazine^b^	Pimozide^b^	Pipamperone^b^	Prochlorperazine^b^	Propericiazine^b^	Reserpine^b^
Altered state of consciousness	0.0000	5.5556	1.0101	0.0000	0.0000	1.2712	5.3030	0.0000
Autonomic nervous system imbalance	0.0000	0.0000	1.0101	0.0000	0.0000	0.0000	0.0000	0.0000
Blood creatine phosphokinase abnormal	0.0000	0.0000	0.0000	0.0000	0.0000	0.0000	0.0000	0.0000
Blood creatine phosphokinase increased	0.0000	0.0000	3.0303	1.6393	0.0000	1.6949	3.0303	0.0000
Blood pressure abnormal	0.0000	0.0000	0.0000	0.0000	0.0000	0.0000	0.0000	0.0000
Blood pressure decreased	0.0000	0.0000	0.0000	0.0000	0.0000	0.0000	2.2727	5.5556
Blood pressure fluctuation	0.0000	0.0000	0.0000	0.0000	0.0000	0.0000	0.0000	0.0000
Blood pressure increased	0.0000	0.0000	1.0101	0.0000	0.0000	0.0000	0.0000	0.0000
Body temperature increased	0.0000	0.0000	0.0000	0.0000	0.0000	0.0000	0.0000	0.0000
Catatonia	0.0000	0.0000	0.0000	0.0000	0.0000	0.0000	0.0000	0.0000
Coma	0.0000	0.0000	1.0101	0.0000	0.0000	0.4237	0.7576	0.0000
Confusional state	0.0000	0.0000	3.0303	0.0000	0.0000	0.8475	0.0000	0.0000
Delirium	0.0000	0.0000	1.0101	0.0000	0.0000	6.7797	3.0303	0.0000
Depressed level of consciousness	0.0000	0.0000	0.0000	0.0000	0.0000	0.4237	0.7576	0.0000
Disorientation	0.0000	0.0000	0.0000	0.0000	0.0000	0.0000	0.0000	0.0000
Dyskinesia	0.0000	0.0000	4.0404	1.6393	0.0000	0.8475	0.7576	0.0000
Dystonia	0.0000	5.5556	5.0505	16.3934	0.0000	0.8475	6.8182	0.0000
Extrapyramidal disorder	0.0000	0.0000	3.0303	0.0000	4.7619	5.9322	1.5152	0.0000
Heart rate increased	0.0000	0.0000	0.0000	0.0000	0.0000	0.0000	0.0000	0.0000
Hyperhidrosis	0.0000	0.0000	1.0101	0.0000	0.0000	0.8475	0.7576	0.0000
Hyperkinesia	0.0000	0.0000	0.0000	0.0000	0.0000	0.0000	0.0000	0.0000
Hyperpyrexia	0.0000	0.0000	0.0000	0.0000	0.0000	0.0000	0.0000	0.0000
Hypertension	6.2500	0.0000	0.0000	0.0000	0.0000	0.0000	0.0000	0.0000
Hyperthermia malignant	0.0000	0.0000	0.0000	0.0000	0.0000	0.0000	0.0000	0.0000
Hypertonia	0.0000	0.0000	0.0000	0.0000	0.0000	0.0000	0.0000	0.0000
Hypotension	0.0000	0.0000	1.0101	0.0000	0.0000	0.4237	2.2727	5.5556
Labile blood pressure	0.0000	0.0000	0.0000	0.0000	0.0000	0.0000	0.0000	0.0000
Leukocytosis	0.0000	0.0000	0.0000	0.0000	0.0000	0.0000	0.0000	0.0000
Loss of consciousness	0.0000	0.0000	5.0505	0.0000	0.0000	0.8475	1.5152	0.0000
Muscle rigidity	0.0000	0.0000	0.0000	0.0000	0.0000	0.0000	0.0000	0.0000
Myoclonus	0.0000	0.0000	0.0000	0.0000	0.0000	0.4237	0.0000	0.0000
Myoglobin blood increased	0.0000	0.0000	0.0000	0.0000	0.0000	0.0000	0.0000	0.0000
Myoglobinuria	0.0000	0.0000	0.0000	0.0000	0.0000	0.0000	0.0000	0.0000
Myoglobin urine present	0.0000	0.0000	0.0000	0.0000	0.0000	0.0000	0.0000	0.0000
Neuroleptic malignant syndrome	25.0000	16.6667	17.1717	14.7541	9.5238	4.2373	17.4242	0.0000
Oculogyric crisis	0.0000	0.0000	0.0000	0.0000	0.0000	0.4237	0.0000	0.0000
Opisthotonus	0.0000	0.0000	0.0000	0.0000	0.0000	0.0000	0.0000	0.0000
Parkinsonism	0.0000	5.5556	8.0808	6.5574	0.0000	2.5424	0.7576	5.5556
Pyrexia	0.0000	0.0000	2.0202	0.0000	4.7619	2.5424	3.0303	0.0000
Rhabdomyolysis	0.0000	0.0000	3.0303	0.0000	14.2857	2.5424	6.0606	5.5556
Serotonin syndrome	0.0000	0.0000	0.0000	0.0000	0.0000	1.6949	0.0000	0.0000
Slow response to stimuli	0.0000	0.0000	0.0000	0.0000	0.0000	0.0000	0.0000	0.0000
Stupor	0.0000	0.0000	1.0101	0.0000	0.0000	0.0000	0.0000	0.0000
Tachycardia	0.0000	0.0000	0.0000	0.0000	0.0000	0.0000	0.0000	5.5556
Tremor	0.0000	0.0000	0.0000	0.0000	0.0000	2.9661	0.0000	0.0000
Unresponsive to stimuli	0.0000	0.0000	0.0000	0.0000	0.0000	0.0000	0.0000	0.0000
White blood cell count increased	0.0000	0.0000	1.0101	0.0000	0.0000	0.0000	0.7576	0.0000
Consciousness fluctuating	0.0000	0.0000	0.0000	0.0000	0.0000	0.0000	0.0000	0.0000
Muscle enzyme increased	0.0000	0.0000	0.0000	0.0000	0.0000	0.0000	0.0000	0.0000
Freezing phenomenon	0.0000	0.0000	0.0000	0.0000	0.0000	0.0000	0.0000	0.0000
Parkinson's disease	0.0000	0.0000	0.0000	0.0000	0.0000	1.2712	0.0000	0.0000
Cardiovascular insufficiency	0.0000	0.0000	0.0000	0.0000	0.0000	0.0000	0.0000	0.0000
Malignant catatonia	0.0000	0.0000	0.0000	0.0000	0.0000	0.0000	0.0000	0.0000
Withdrawal catatonia	0.0000	0.0000	0.0000	0.0000	0.0000	0.0000	0.0000	0.0000

**Table 5:** Reporting ratios (%) of preferred term of neuroleptic malignant syndrome associated with antipsychotics (continued). ^a^Source: References [17, 18] ^b^TAP: typical antipsychotic ^c^AAP: atypical antipsychotic

Preferred term^a^	Spiperone^b^	Sultopride^b^	Tiapride^b^	Timiperone^b^	Zotepine^b^	Aripiprazole^c^	Asenapine^c^	Blonanserin^c^
Altered state of consciousness	0.0000	1.1628	4.7423	0.0000	2.2680	1.9374	1.8149	0.9582
Autonomic nervous system imbalance	0.0000	0.0000	0.0000	0.0000	0.0000	0.0000	0.0000	0.0000
Blood creatine phosphokinase abnormal	0.0000	0.0000	0.0000	0.0000	0.0000	0.0497	0.0000	0.0000
Blood creatine phosphokinase increased	0.0000	2.3256	1.4433	2.4390	2.4742	3.6761	1.4519	2.7003
Blood pressure abnormal	0.0000	0.0000	0.0000	0.0000	0.0000	0.0000	0.0000	0.0000
Blood pressure decreased	0.0000	0.0000	2.0619	0.0000	0.6186	0.3974	1.0889	0.6098
Blood pressure fluctuation	0.0000	0.0000	0.2062	0.0000	0.0000	0.0000	0.0000	0.0000
Blood pressure increased	0.0000	0.0000	0.2062	0.0000	0.0000	0.0745	0.1815	0.1742
Body temperature increased	0.0000	0.0000	0.0000	0.0000	0.0000	0.0248	0.0000	0.0871
Catatonia	0.0000	0.0000	0.0000	0.0000	0.6186	0.1490	0.0000	0.1742
Coma	0.0000	0.0000	1.6495	0.0000	0.0000	0.1739	0.1815	0.5226
Confusional state	0.0000	0.0000	0.0000	0.0000	0.0000	0.1242	0.0000	0.1742
Delirium	0.0000	0.0000	2.2680	0.0000	0.2062	0.7948	0.0000	0.0871
Depressed level of consciousness	0.0000	2.3256	2.0619	2.4390	0.6186	0.4223	0.7260	0.3484
Disorientation	0.0000	0.0000	0.2062	0.0000	0.0000	0.0000	0.1815	0.0000
Dyskinesia	0.0000	4.6512	2.0619	2.4390	1.2371	1.0929	0.7260	1.5679
Dystonia	0.0000	4.6512	2.0619	12.1951	4.7423	3.4526	1.6334	3.3101
Extrapyramidal disorder	0.0000	0.0000	1.0309	2.4390	1.6495	1.3413	1.4519	2.7875
Heart rate increased	0.0000	0.0000	0.0000	0.0000	0.0000	0.0745	0.0000	0.0871
Hyperhidrosis	0.0000	0.0000	0.0000	0.0000	0.2062	0.1987	0.1815	0.0871
Hyperkinesia	0.0000	0.0000	0.0000	0.0000	0.2062	0.1739	0.0000	0.0871
Hyperpyrexia	0.0000	0.0000	0.0000	0.0000	0.0000	0.0248	0.0000	0.0871
Hypertension	0.0000	1.1628	0.0000	0.0000	0.0000	0.3229	0.0000	0.0871
Hyperthermia malignant	0.0000	0.0000	0.0000	0.0000	0.0000	0.0000	0.0000	0.0000
Hypertonia	0.0000	0.0000	0.2062	0.0000	0.0000	0.0248	0.0000	0.0000
Hypotension	0.0000	0.0000	0.8247	0.0000	0.0000	0.2981	0.5445	0.5226
Labile blood pressure	0.0000	0.0000	0.0000	0.0000	0.0000	0.0000	0.0000	0.0000
Leukocytosis	0.0000	0.0000	0.0000	0.0000	0.0000	0.0000	0.0000	0.0000
Loss of consciousness	0.0000	0.0000	2.0619	0.0000	0.8247	0.9687	1.8149	0.3484
Muscle rigidity	0.0000	0.0000	0.0000	0.0000	0.2062	0.3229	0.5445	0.7840
Myoclonus	0.0000	0.0000	0.0000	0.0000	0.0000	0.0745	0.0000	0.0000
Myoglobin blood increased	0.0000	0.0000	0.0000	0.0000	0.0000	0.0248	0.0000	0.0000
Myoglobinuria	0.0000	0.0000	0.0000	0.0000	0.0000	0.0248	0.0000	0.0000
Myoglobin urine present	0.0000	0.0000	0.0000	0.0000	0.0000	0.0248	0.0000	0.0000
Neuroleptic malignant syndrome	50.0000	47.6744	13.1959	21.9512	28.2474	10.7303	10.8893	21.5157
Oculogyric crisis	0.0000	0.0000	0.0000	0.0000	0.4124	0.7700	0.9074	1.2195
Opisthotonus	0.0000	0.0000	0.0000	0.0000	0.0000	0.0248	0.0000	0.0871
Parkinsonism	0.0000	0.0000	3.0928	0.0000	2.0619	1.8629	2.3593	1.5679
Pyrexia	0.0000	1.1628	2.0619	2.4390	1.6495	0.9190	0.7260	0.5226
Rhabdomyolysis	0.0000	1.1628	3.7113	0.0000	3.9175	4.2722	4.7187	7.0557
Serotonin syndrome	0.0000	0.0000	0.2062	0.0000	0.0000	0.5464	0.0000	0.0000
Slow response to stimuli	0.0000	0.0000	0.0000	0.0000	0.0000	0.0000	0.0000	0.0000
Stupor	0.0000	0.0000	0.0000	0.0000	0.0000	0.6706	0.9074	0.3484
Tachycardia	0.0000	0.0000	0.2062	0.0000	0.2062	0.1490	0.0000	0.0871
Tremor	0.0000	0.0000	0.8247	0.0000	0.8247	0.5216	0.9074	0.8711
Unresponsive to stimuli	0.0000	0.0000	0.4124	0.0000	0.0000	0.0248	0.0000	0.0000
White blood cell count increased	0.0000	0.0000	0.0000	0.0000	0.0000	0.1987	0.1815	0.0000
Consciousness fluctuating	0.0000	0.0000	0.0000	0.0000	0.0000	0.0248	0.0000	0.0000
Muscle enzyme increased	0.0000	0.0000	0.0000	0.0000	0.0000	0.0000	0.0000	0.0000
Freezing phenomenon	0.0000	0.0000	0.0000	0.0000	0.0000	0.0000	0.0000	0.0871
Parkinson's disease	0.0000	0.0000	0.4124	0.0000	0.0000	0.3726	0.0000	0.0000
Cardiovascular insufficiency	0.0000	0.0000	0.0000	0.0000	0.0000	0.0000	0.0000	0.0000
Malignant catatonia	0.0000	0.0000	0.0000	0.0000	0.4124	0.2732	0.0000	0.0871
Withdrawal catatonia	0.0000	0.0000	0.0000	0.0000	0.0000	0.0000	0.0000	0.0000

**Table 6:** Reporting ratios (%) of preferred term of neuroleptic malignant syndrome associated with antipsychotics. ^a^Source: References [17, 18] ^b^TAP: typical antipsychotic ^c^AAP: atypical antipsychotic

Preferred term^a^	Brexpiprazole^c^	Clozapine^c^	Lurasidone^c^	Olanzapine^c^	Paliperidone^c^	Perospirone^c^	Quetiapine^c^	Risperidone^c^
Altered state of consciousness	1.8739	0.6919	0.3344	2.0756	1.3272	1.3491	2.1196	2.7572
Autonomic nervous system imbalance	0.0000	0.0000	0.0000	0.0000	0.0000	0.0000	0.0581	0.0000
Blood creatine phosphokinase abnormal	0.0000	0.0000	0.0000	0.0310	0.0000	0.0000	0.0000	0.0000
Blood creatine phosphokinase increased	4.2589	0.9410	1.0033	2.1685	1.0964	2.5295	1.5679	2.3537
Blood pressure abnormal	0.0000	0.0000	0.0000	0.0000	0.0000	0.0000	0.0000	0.0000
Blood pressure decreased	0.0000	0.4428	0.1672	0.6506	0.4039	0.3373	0.6678	0.7173
Blood pressure fluctuation	0.0000	0.0277	0.0000	0.0000	0.0000	0.0000	0.0871	0.0000
Blood pressure increased	0.3407	0.5812	0.0000	0.0620	0.3462	0.5059	0.1742	0.1793
Body temperature increased	0.0000	0.0000	0.0000	0.0000	0.0000	0.0000	0.0000	0.0000
Catatonia	0.0000	0.1107	0.0000	0.3408	0.1731	0.3373	0.0581	0.3587
Coma	0.6814	0.0277	0.0000	0.2169	0.0577	0.5059	0.6098	0.4707
Confusional state	0.1704	0.0554	0.3344	0.0929	0.1154	0.0000	0.1742	0.0897
Delirium	0.6814	0.3875	0.5017	1.0223	0.1154	0.5059	1.7422	0.8742
Depressed level of consciousness	1.0221	0.8027	0.3344	0.7435	0.7501	2.1922	1.6551	1.2553
Disorientation	0.0000	0.0554	0.0000	0.0310	0.0000	0.0000	0.0000	0.0448
Dyskinesia	1.1925	0.1107	0.6689	1.0843	0.8078	3.8786	1.1324	1.4122
Dystonia	3.5775	0.1384	10.3679	2.7261	1.9619	7.9258	2.2938	3.1383
Extrapyramidal disorder	1.3629	0.3598	0.1672	1.6419	2.6544	3.0354	0.7259	1.7709
Heart rate increased	0.0000	0.4428	0.0000	0.0000	0.0000	0.1686	0.0290	0.0672
Hyperhidrosis	0.1704	0.0000	0.0000	0.1239	0.0577	0.1686	0.2033	0.0897
Hyperkinesia	0.0000	0.0000	0.0000	0.0310	0.0000	0.0000	0.0000	0.0448
Hyperpyrexia	0.0000	0.0000	0.0000	0.0000	0.0000	0.0000	0.0000	0.0000
Hypertension	0.0000	0.6643	0.0000	0.0929	0.1154	0.0000	0.1452	0.1121
Hyperthermia malignant	0.0000	0.0277	0.0000	0.0310	0.0000	0.0000	0.0000	0.0672
Hypertonia	0.0000	0.0000	0.0000	0.0000	0.0000	0.0000	0.0000	0.0000
Hypotension	0.1704	0.1384	0.0000	0.2788	0.2885	0.0000	0.6388	0.4035
Labile blood pressure	0.0000	0.0000	0.0000	0.0620	0.0000	0.0000	0.0000	0.0000
Leukocytosis	0.0000	0.0554	0.0000	0.0000	0.0000	0.1686	0.0000	0.0000
Loss of consciousness	0.5111	0.6089	1.5050	0.8984	0.9810	0.6745	1.0453	1.0984
Muscle rigidity	0.8518	0.0554	0.0000	0.1859	0.2308	0.3373	0.2613	0.2017
Myoclonus	0.0000	0.4705	0.0000	0.0929	0.0577	0.0000	0.0000	0.0448
Myoglobin blood increased	0.0000	0.0000	0.0000	0.0310	0.0000	0.0000	0.0000	0.0000
Myoglobinuria	0.0000	0.0000	0.0000	0.0000	0.0000	0.0000	0.0000	0.0448
Myoglobin urine present	0.0000	0.0000	0.0000	0.0310	0.0000	0.0000	0.0290	0.0224
Neuroleptic malignant syndrome	12.6065	2.2419	2.1739	13.9715	7.3860	18.3811	9.9303	14.1000
Oculogyric crisis	3.5775	0.0277	6.6890	0.2169	2.5389	0.8432	0.2613	0.9191
Opisthotonus	0.0000	0.0000	0.0000	0.0000	0.0000	0.0000	0.0000	0.0224
Parkinsonism	1.7036	0.3598	0.6689	1.6419	1.4426	4.3845	1.2776	2.4882
Pyrexia	1.0221	1.6883	0.1672	0.8055	0.9233	1.0118	1.0163	1.0312
Rhabdomyolysis	5.1107	0.9964	1.1706	3.9963	3.1160	4.2159	2.8746	5.7835
Serotonin syndrome	0.8518	0.0000	0.1672	0.4647	0.0577	1.8550	0.6969	0.3587
Slow response to stimuli	0.0000	0.0000	0.0000	0.0310	0.0000	0.0000	0.0000	0.0000
Stupor	0.3407	0.1937	0.0000	0.2169	0.2885	0.5059	0.0581	0.2242
Tachycardia	0.0000	1.7437	0.0000	0.1549	0.1731	0.5059	0.1742	0.1793
Tremor	0.3407	0.1107	0.1672	0.4957	0.4039	0.6745	0.5517	0.6501
Unresponsive to stimuli	0.0000	0.0277	0.0000	0.0310	0.0577	0.0000	0.0581	0.0000
White blood cell count increased	0.1704	0.3875	0.0000	0.1239	0.0577	0.0000	0.0581	0.1121
Consciousness fluctuating	0.0000	0.0277	0.0000	0.0000	0.0000	0.0000	0.0290	0.0000
Muscle enzyme increased	0.0000	0.0277	0.0000	0.0000	0.0000	0.0000	0.0000	0.0000
Freezing phenomenon	0.0000	0.0277	0.0000	0.0000	0.0000	0.0000	0.0000	0.0000
Parkinson's disease	0.5111	0.1384	0.5017	0.1239	0.2885	0.0000	0.1161	0.3362
Cardiovascular insufficiency	0.0000	0.0000	0.0000	0.0000	0.0000	0.0000	0.0000	0.0672
Malignant catatonia	0.1704	0.0277	0.0000	0.2169	0.1154	0.0000	0.1452	0.3587
Withdrawal catatonia	0.0000	0.0277	0.0000	0.0000	0.0000	0.0000	0.0581	0.0000
